# Brain gray matter network organization in psychotic disorders

**DOI:** 10.1038/s41386-019-0586-2

**Published:** 2019-12-07

**Authors:** Wenjing Zhang, Du Lei, Sarah K. Keedy, Elena I. Ivleva, Seenae Eum, Li Yao, Carol A. Tamminga, Brett A. Clementz, Matcheri S. Keshavan, Godfrey D. Pearlson, Elliot S. Gershon, Jeffrey R. Bishop, Qiyong Gong, Su Lui, John A. Sweeney

**Affiliations:** 1 Huaxi MR Research Center (HMRRC), Department of Radiology, Functional and Molecular Imaging Key Laboratory of Sichuan Province, West China Hospital, Sichuan University, Chengdu, China; 2 Department of Psychiatry and Behavioral Neuroscience, University of Cincinnati, Cincinnati, OH USA; 3 Department of Psychiatry and Behavioral Neuroscience, University of Chicago, Chicago, IL USA; 4 Department of Psychiatry, University of Texas Southwestern Medical Center, Dallas, TX USA; 5 Department of Experimental and Clinical Pharmacology, College of Pharmacy, University of Minnesota, Minneapolis, MN USA; 6 Department of Psychology, University of Georgia, Athens, GA USA; 7 Department of Psychiatry, Beth Israel Deaconess Medical Center, Harvard Medical School, Boston, MA USA; 8 Department of Psychiatry, School of Medicine, Yale University, New Haven, CT USA

**Keywords:** Psychosis, Cognitive neuroscience

## Abstract

Abnormal neuroanatomic brain networks have been reported in schizophrenia, but their characterization across patients with psychotic disorders, and their potential alterations in nonpsychotic relatives, remain to be clarified. Participants recruited by the Bipolar and Schizophrenia Network for Intermediate Phenotypes consortium included 326 probands with psychotic disorders (107 with schizophrenia (SZ), 87 with schizoaffective disorder (SAD), 132 with psychotic bipolar disorder (BD)), 315 of their nonpsychotic first-degree relatives and 202 healthy controls. Single-subject gray matter graphs were extracted from structural MRI scans, and whole-brain neuroanatomic organization was compared across the participant groups. Compared with healthy controls, psychotic probands showed decreased nodal efficiency mainly in bilateral superior temporal regions. These regions had altered morphological relationships primarily with frontal lobe regions, and their network-level alterations were associated with positive symptoms of psychosis. Nonpsychotic relatives showed lower nodal centrality metrics in the prefrontal cortex and subcortical regions, and higher nodal centrality metrics in the left cingulate cortex and left thalamus. Diagnosis-specific analysis indicated that individuals with SZ had lower nodal efficiency in bilateral superior temporal regions than controls, probands with SAD only exhibited lower nodal efficiency in the left superior and middle temporal gyrus, and individuals with psychotic BD did not show significant differences from healthy controls. Our findings provide novel evidence of clinically relevant disruptions in the anatomic association of the superior temporal lobe with other regions of whole-brain networks in patients with psychotic disorders, but not in their unaffected relatives, suggesting that it is a disease-related trait. Network disorganization primarily involving frontal lobe and subcortical regions in nonpsychotic relatives may be related to familial illness risk.

## Introduction

Efforts to identify imaging markers for psychotic disorders have been impeded by two factors: a focus on traditionally defined psychotic syndromes that have overlapping genetic, psychological, and neurobiological features [1–3], and a focus on regional changes such as gray matter volume [4, 5] or brain activity [6], or altered functional connectivity between pairs of regions [7, 8], rather than comprehensively on whole-brain networks that are believed to be fundamentally involved in the pathogenesis of psychosis [9–11]. Furthermore, prior studies in this area often utilized relatively small samples or post-hoc analyses of large data sets not collected with rigorous attention to consistency of image acquisition across ascertainment sites.

Phenotyping approaches grounded in network science can provide a more comprehensive understanding of neuropathological substrates of psychosis [10, 12, 13]. Graph theory approaches based on functional connectivity and white matter connectivity have been used to characterize network graphs in patients with psychotic disorders [12, 14–17] and their relatives [18, 19]. However, the blood oxygen level-dependent (BOLD) signals reflecting brain function depend greatly on brain states [20, 21], and measurement of white matter pathways is affected by selection of tractography algorithms [22]. Approaches using brain gray matter anatomy to investigate brain networks in psychotic disorders may reveal more stable phenotypes related to altered anatomical organization [23–26]. The potential importance of this type of analysis for evaluating behaviorally relevant features of brain anatomy is reflected in observations that individual variation in morphometric similarity networks can account for ~40% of the individual differences in IQ scores in healthy young people [25].

Previous studies characterized structural gray matter “connections” in schizophrenia patients [27, 28] and their offspring [29, 30] based on covariation in cortical gray matter measures such as volume or cortical thickness across the brain. Individuals with schizophrenia demonstrated a network abnormality with temporal rather than frontal hubs [27], and unaffected family members showed increased gray matter correlations within default-mode network regions [29]. However, in these analyses, gray matter networks were calculated by creating a whole-brain network for each group, thus individual networks for each participant could not be examined and related to clinical parameters of interest. An additional methodological issue is that studies mapping individual brains into standard space before characterizing anatomic networks might have lost precision in quantitation of network topology from not taking the complex individualized folding structure of the brain into account. Notably, a recent study investigating the morphometric similarity across brain regions in patients with nonaffective psychoses demonstrated altered morphometric similarity in the frontal and temporal cortex, which were associated with brain expression of schizophrenia-related genes [23]. These previous findings support the potential of investigating anatomical brain networks across psychotic disorders and in nonpsychotic family members.

In this study, we generated single-subject gray matter graphs in psychotic individuals and their first-degree relatives with no history of psychosis collected by the Bipolar and Schizophrenia Network for Intermediate Phenotypes (B-SNIP) consortium. The primary analysis considered psychotic disorder patients combined across DSM diagnoses, based on growing evidence of overlapping cognitive, imaging, and genetic alterations across these conditions [31–34]. Secondary analyses were conducted for patients with each DSM diagnosis and their relatives.

## Materials and methods

### Participants

The B-SNIP consortium recruited participants across five sites in the United States that carried out parallel recruitment and phenotyping procedures as described previously [35]. We included 843 participants: 326 psychosis probands (107 with schizophrenia (SZ), 87 with schizoaffective disorder (SAD), and 132 with psychotic bipolar I disorder (BD)), 315 of their nonpsychotic first-degree relatives (120 of individuals with SZ, 83 of individuals with SAD, and 112 of individuals with psychotic BD), and 202 healthy participants (Table 1; Supplementary Table S1). Of patient probands included, all with artifact-free scans, 47.2% had at least one family member who had a MR scan performed and whose data met quality control (QC) standards discussed below. These relatives and their patient proband relative were included in within-family analyses described below. The study was approved by the Institutional Review Boards at each site, and all participants provided written informed consent prior to participation.

**Table 1:** Demographic and clinical parameters for psychosis probands, their nonpsychotic relatives, and healthy controls.

	Probands (*N* = 326)	Relatives (*N* = 315)	Healthy controls (*N* = 202)	F	*p*
	Mean (standard deviation)				
Age (years)	35.35 (12.45)	39.83 (15.68)	36.59 (12.55)	8.85	<0.001
Education	13.59 (2.41)	14.32 (2.64)	14.96 (2.39)	19.25	<0.001
GAF	53.50 (13.96)	77.08 (12.39)	86.65 (6.54)	555.03	<0.001
WRAT	100.22 (15.13)	101.25 (15.03)	103.64 (14.10)	3.36	0.035
BACS	−1.19 (1.40)	−0.32 (1.21)	0.02 (1.11)	67.69	<0.001
PANSS_Total	64.21 (17.42)				
PANSS_Positive	16.33 (5.53)				
PANSS_Negative	14.81 (5.44)				
YMRS_Total	6.47 (6.27)				
MADRS_Total	11.38 (9.33)				
CPZ dose (mg/day)	406.31 (381.37)				

	*N* (%)	*N* (%)	*N* (%)	χ^2^	*P*
Sex (male, %)	143 (43.9%)	91 (28.9%)	88 (43.6%)	18.46	<0.001
Handed (right, %)	273 (83.7%)	272 (86.3%)	177 (87.6%)	2.48	0.648
*Race*					
Caucasian	190 (58.3%)	214 (67.9%)	134 (66.3%)	12.19	0.016
African American	115 (35.3%)	86 (27.3%)	50 (24.8%)		
Other	21 (6.4%)	15 (4.8%)	18 (8.9%)		

*BACS* Brief Assessment of Cognition in Schizophrenia (z-score), *CPZ* Chlorpromazine Equivalent Antipsychotic Dosage, *GAF* Global Assessment of Functioning, *MADRS* Montgomery-Åsberg Depression Rating Scale, *PANSS* Positive and Negative Syndrome Scale, *WRAT* wide-range achievement test, *YMRS* Young Mania Rating Scale

Probands were clinically stable and receiving consistent pharmacological treatment over the preceding month. The relatives included had no history of psychotic disorders. Axis I diagnoses in all groups were made based on the Structured Clinical Interview for DSM-IV Axis I Disorders and consensus case review. Diagnoses of Axis II disorders in relatives were made using the Structured Interview for DSM-IV Personality Disorders (SIDP-IV). Exclusion criteria for all participants included: (1) contraindication to MRI scans, (2) substance abuse within past 1 month or substance dependence within 3 months, (3) significant systemic or neurological disorder, and (4) history of significant head trauma. Details regarding recruitment and clinical assessment strategies used by the B-SNIP consortium are available [33, 35].

### MRI acquisition

High-resolution T1-weighted images were acquired using 3.0 T MRI scanners following guidelines of the Alzheimer’s Disease Neuroimaging Initiative (ADNI) protocol (http://www.loni.ucla.edu/ADNI/Research/Cores/) to enhance image consistency across sites/scanners (Supplementary Table S2). Quantitative image quality ratings (IQR) were calculated for each subject and did not differ across participant groups (details in Supplementary Materials).

### Extraction of brain networks

We followed the methodology proposed by Tijms et al. [36] to extract individual structural morphology brain networks, which is a completely automated and data-driven method. This approach takes both local morphology (e.g., thickness) and folding structure of the cortex into account, improving accuracy in the characterization of morphological network topology [24, 37]. In brief, the method defines network nodes as regions of interest (ROI) corresponding to 3 × 3 × 3 mm^3^ voxel cubes, and their “connection” refers to “edges”, indicating statistically similar gray matter morphology of two cubes as determined with correlation coefficients. Weighted graphs are constructed after determining a threshold for each individual graph with a permutation-based method to ensure a significant similarity (*P* < 0.05) for all individuals [38]. Because network properties can vary with network size [39], we normalized gray matter networks using the methodology proposed by Batalle et al. [40] based on the Automated Anatomical Labeling (AAL) parcellation template [41] (details in Supplementary Materials).

### Network properties

GRETNA (www.nitrc.org/projects/gretna/) was used to calculate topological properties of brain networks. A wide range of sparsity (S) thresholds was applied to all correlation matrices. S was determined to ensure that thresholded networks were estimable for the small-worldness scalar (σ), defined as σ > 1.0. Our threshold range was 0.05–0.40 with an interval of 0.01. This was determined using two criteria: (1) the average node of each threshold network on all nodes degree (node degree refers to the number of all edges connected to a node) is >2 × log (*N*) (where *N* is 90, indicating the number of nodes); and (2) small-world scalar σ of the threshold network of all subjects (as defined below) is >1.1 [42].

Global and nodal network properties were calculated at each sparsity threshold. Then, the area under the curve (AUC) was calculated for each network metric, providing a summarizing scalar for characterizing topological properties of brain networks independent of any single threshold selection as in previous studies [43, 44]. The following global metrics of small-world parameters were examined [42]: clustering coefficient (C_p_), characteristic path length (L_p_), normalized clustering coefficient (γ), normalized characteristic path length (λ), and small worldness (σ). Network efficiency parameters, including local efficiency (E_loc_) and global efficiency (E_glob_) [45], nodal centrality metrics including degree, efficiency, and betweenness, were also examined. Detailed explanation of each parameter is provided in Supplementary Materials.

Because the default-mode network (DMN), central executive network (CEN) and salience network (SN) have been shown to be altered in psychosis probands and their relatives [46–49], their gray matter topological network metrics were also calculated and compared among participant groups (Supplementary Materials).

### Statistical analysis

#### Group comparison of network metrics

Analyses of covariance (ANCOVA) were performed to test for group differences in each network metrics (small-world, network efficiency, and nodal centrality measures) separately in psychosis probands and their nonpsychotic relatives in comparison with healthy controls. Site, age, sex, race, and handedness were included as covariates, and analyses used a false discovery rate (FDR) correction to preserve a *P* < 0.05 experiment-wise threshold.

Abnormal network metrics found to be abnormal either in patient probands or relatives were examined separately in relation to DSM diagnoses of patient probands and experimental psychosis Biotypes defined by the B-SNIP consortium using a data-driven approach and cognitive, eye movement, and electroencephalographic (EEG) measures [3]. Findings from these analyses are shown in Supplementary Materials.

#### Within-family correlations

After excluding patient and relative cases based on QC of the MRI data or relatives being unwilling or unable to have MRI scans, 47% of the patient probands had a family member with valid MRI data. To analyze patients and relatives together considering their family relationship, we included these patients and relatives and the controls with ANCOVAs as above to identify group differences, treating family membership as a random effect. Second, we examined the correlation of the data from these patients and their specific relatives, and within diagnoses for all global network measures and all metrics in which differences were seen in comparisons of relatives or probands vs. healthy controls. Site, age, sex, race, and handedness were treated as covariates in these analyses. Findings are shown in Supplementary Materials.

#### Network matrix comparisons of abnormal nodes

The correlation matrices of inter-region associations of regions with altered nodal characteristics were examined to identify regions with altered connection to aberrant nodes found in psychotic probands or their nonpsychotic relatives in comparison with correlations in controls using the network-based statistics (NBS) method [50]. Findings are shown in Supplementary Materials.

#### Correlations with clinical variables

Structural network metrics that differed between probands and controls were correlated with psychiatric symptom severity ratings using the Positive and Negative Syndrome Scale (PANSS) [51], Young Mania Rating Scale (YMRS) [52], Montgomery-Åsberg Depression Rating Scale (MADRS) [53], and the Global Assessment of Functioning (GAF) scale, adjusting for site, age, sex, race, and handedness. We also examined associations with general cognitive functioning as assessed by the Brief Assessment of Cognition in Schizophrenia (BACS) scale [31]. In nonpsychotic relatives, altered network metrics were correlated with the presence of psychosis spectrum (Cluster A) personality features (within 1 criterion of diagnosis). Since the relative sample includes younger individuals still in the risk period, age effects on altered network metrics were also examined. Nominal significance thresholds were used for these exploratory/heuristic analyses.

## Results

### Topological metrics of brain gray matter networks

All patient probands, their nonpsychotic relatives, and healthy controls showed a small-world architecture (i.e., σ > 1) at all connection densities. There were no significant differences in psychosis probands or their nonpsychotic relatives compared with healthy controls in global network properties, including global/local efficiency, clustering coefficient, characteristic path length, normalized clustering coefficient, or normalized characteristic path length.

**Fig. 1:**
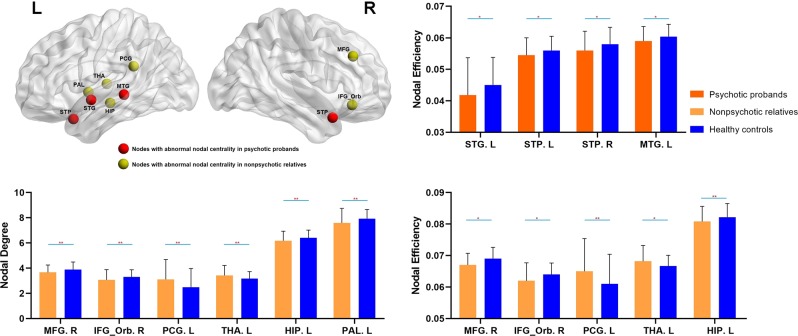
Intergroup comparisons of gray matter network metrics between psychotic probands or their nonpsychotic relatives and healthy controls. **P < 0.01; *P < 0.05. HIP hippocampus, MFG middle frontal gyrus, MTG middle temporal gyrus, IFG_Orb orbital inferior frontal gyrus, PCG posterior cingulate gyrus, PAL pallidum, STG superior temporal gyrus, STP superior temporal pole, THA thalamus, L left, R right.

Relative to healthy controls, probands with psychotic disorders showed reduced nodal efficiency in the left superior temporal gyrus (STG), left middle temporal gyrus (MTG), and bilateral superior temporal pole (STP), implying reduced capacity for propagating information with other brain nodes. Compared with healthy controls, nonpsychotic relatives had significantly lower nodal degree (fewer nodal connections) in the right middle frontal gyrus (MFG), right orbital inferior frontal gyrus (IFG), left hippocampus, left pallidum, and higher nodal degree in the left posterior cingulate gyrus (PCG) and left thalamus. Nonpsychotic relatives also showed significantly lower nodal efficiency in right MFG, right orbital IFG, left hippocampus, and higher nodal efficiency in the left PCG and left thalamus compared with healthy controls (all with FDR corrected *P* < 0.05, Fig. 1, Table 2). All of these findings remained significant after IQR was treated as an additional covariate.

**Table 2:** Network metrics of regions showing significant intergroup differences in psychotic probands and their nonpsychotic relatives.

Regions with altered network metrics	Nodal centrality metrics	Probands (*N* = 326)/relatives (*N* = 315)	HC (*N* = 202)	ANCOVA	
		Mean ± SD	Mean ± SD	F	P*
*Psychotic probands vs healthy controls*					
STG, L	Efficiency	0.042 ± 0.012	0.045 ± 0.009	11.36	0.024
STP, L	Efficiency	0.055 ± 0.006	0.056 ± 0.004	10.37	0.031
STP, R	Efficiency	0.056 ± 0.006	0.058 ± 0.005	11.99	0.024
MTG, L	Efficiency	0.059 ± 0.005	0.060 ± 0.004	9.55	0.038
*Nonpsychotic relatives vs healthy controls*					
MFG, R	Degree	3.67 ± 0.58	3.89 ± 0.60	10.43	0.009
IFG_Orb, R	Degree	3.07 ± 0.81	3.30 ± 0.57	12.93	0.006
PCG, L	Degree	3.11 ± 1.58	2.49 ± 1.49	17.17	0.004
Hippocampus, L	Degree	6.18 ± 0.75	6.40 ± 0.62	12.57	0.006
Pallidum, L	Degree	7.58 ± 1.16	7.93 ± 0.73	12.42	0.006
Thalamus, L	Degree	3.42 ± 0.79	3.17 ± 0.55	12.39	0.006
MFG, R	Efficiency	0.067 ± 0.004	0.069 ± 0.004	9.52	0.013
IFG_Orb, R	Efficiency	0.062 ± 0.006	0.064 ± 0.004	9.07	0.018
PCG, L	Efficiency	0.065 ± 0.010	0.061 ± 0.009	14.12	0.003
Hippocampus, L	Efficiency	0.081 ± 0.005	0.082 ± 0.004	14.31	0.003
Thalamus, L	Efficiency	0.068 ± 0.005	0.067 ± 0.003	9.12	0.018

*ANCOVA* analysis of covariance, *FDR* false discovery rate, *HC* healthy controls, *SD* standard deviation, *MFG* middle frontal gyrus, *MTG* middle temporal gyrus, *IFG_Orb* orbital inferior frontal gyrus, *PCG* posterior cingulate gyrus, *STG* superior temporal gyrus, *STP* superior temporal pole, *L* left, *R* right

^*^P-values that were corrected with FDR

In the subgroup of patients and relatives from the same families, ANCOVAs were performed as above, but treating family membership as a random effect. These analyses identified significant group differences among probands, nonpsychotic relatives, and healthy controls in nodal efficiency in the left STG, left MTG, and bilateral STP, as well as left PCG and left thalamus. Differences in nodal degree were observed in the right orbital IFG, left PCG, and left thalamus. Direct comparison of psychosis probands with their nonpsychotic relatives from same families can be informative regarding familial and disease associations, as their background genetic and environmental factors are more similar than in the general population. Probands showed lower nodal degree/efficiency compared with their relatives in regions where alterations were seen in comparison of probands with healthy controls in the primary analyses (lower nodal efficiency of the left STG, left MTG, and bilateral STP). Also, in this analysis with better control of background familial factors, we identified an additional alteration of higher nodal degree and efficiency in the left thalamus in relatives compared with probands after FDR correction, similar to the differences between relatives and controls.

### Within-family correlations

In regions with altered anatomic similarity metrics of patient probands or relatives, four out of sixteen had significant familial association. Within the four temporal lobe alterations observed in probands, only the left STG showed modestly significant familiality. In the 11 alterations observed in relatives, nodal degree of the left thalamus and nodal efficiency of the left hippocampus and left thalamus showed significant familiality (Table 3). Disorder-specific analyses indicated that two of these measures were significant in SAD patients, one of these in SZ patients as well, and none in bipolar patients. None of the four temporal lobe metrics altered in patient probands showed significant familiality in any DSM disorder. Detailed findings are shown in Supplementary Materials.

**Table 3:** Within-family correlation of network metrics that had significant intergroup differences among the participant groups.

Regions with altered network metrics	Nodal centrality metrics	Overall probands	DSM disorders		
			SZ	SAD	BD
*Network metrics altered in psychotic probands*					
STG, L	Efficiency	*r* = 0.25, *P* **=** 0.009	*/*	*/*	*/*
STP, L	Efficiency	*/*	*/*	*/*	*/*
STP, R	Efficiency	*/*	*/*	*/*	*/*
MTG, L	Efficiency	*/*	*/*	*/*	*/*
*Network metrics altered in nonpsychotic relatives*					
MFG, R	Degree	*/*	*/*	*/*	*/*
IFG_Orb, R	Degree	*/*	*/*	*/*	*/*
PCG, L	Degree	*/*	*/*	*/*	*/*
Hippocampus, L	Degree	*/*	*/*	*/*	*/*
Pallidum, L	Degree	*/*	*/*	*/*	*/*
Thalamus, L	Degree	*r* = 0.39, *P* **<** 0.001	*/*	*r* = 0.55, *P* **=**0.002	*/*
MFG, R	Efficiency	*/*	*/*	*/*	*/*
IFG_Orb, R	Efficiency	*/*	*/*	*/*	*/*
PCG, L	Efficiency	*/*	*/*	*/*	*/*
Hippocampus, L	Efficiency	*r* = 0.23, *P* **=** 0.018	*/*	*/*	*/*
Thalamus, L	Efficiency	*r* = 0.41, *P* **<** 0.001	*r* = 0.44, *P* **=** 0.017	*r* = 0.58, *P* **=** 0.001	*/*

*BD* psychotic bipolar I disorder, *FDR* false discovery rate, *SAD* schizoaffective disorder, *SZ* schizophrenia, *MFG* middle frontal gyrus, *MTG* middle temporal gyrus, *IFG_Orb* orbital inferior frontal gyrus, *PCG* posterior cingulate gyrus, *STG* superior temporal gyrus, *STP* superior temporal pole, *L* left, *R* right

^*^P-values listed in the table were corrected using FDR

### Network matrix of abnormal nodes

Inspection of correlations of abnormal temporal lobe areas with other brain regions indicated atypical anatomic similarity profiles with prefrontal regions and several subcortical regions relative to the correlations of these brain regions in the healthy controls (Supplementary Table S4). Similar analyses were conducted in regions with altered anatomic similarity profiles for the relatives (Supplementary Table S5).

### Comparisons of altered network metrics in patient diagnostic subgroups

**Fig. 2:**
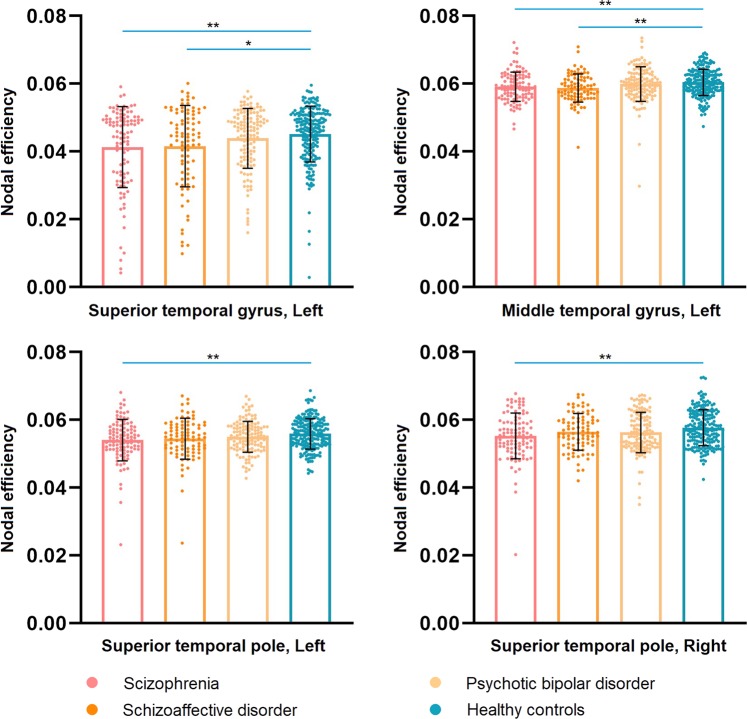
The pair-wise comparisons of selected nodal metrics shown to be altered in the whole proband group between individuals with each DSM diagnosis and healthy controls. **P < 0.01; *P < 0.05.

Network metrics found to be abnormal in the whole proband group were compared separately for each proband DSM diagnostic group vs. controls. Individuals with SZ showed lower nodal efficiency in the left STG, left MTG, and bilateral STP. Probands with SAD exhibited lower nodal efficiency in the left STG and left MTG. Individuals with psychotic BD did not differ from healthy controls. These findings were significant after FDR correction (Fig. 2; Supplementary Table S6). While this pattern is consistent with continuum models of psychotic disorder severity from SZ to SAD to BD, we note that no significant differences were observed among patient groups with different DSM diagnoses across network metrics. Alter network metrics in patients sorted by previously reported B-SNIP experimental Biotypes defined by cognitive and neurophysiological parameters from the same overall patient sample are presented in Supplementary Materials (Supplementary Table S7). Notably, the group with the greatest cognitive and neurophysiological alteration (Biotype 1) had the most significant alterations in temporal lobe metrics. In direct comparison of patients with different Biotypes, no significant group differences were observed.

### Correlation with clinical ratings

In exploratory analyses conducted with nominal significance thresholds, reductions in nodal efficiency of the left STG and bilateral STP were associated with higher PANSS-positive symptom scores in psychosis probands, and reductions in nodal efficiency of left MTG were associated with higher PANSS-positive symptom scores and lower GAF scores (Supplementary Fig. S1). No associations of temporal lobe nodal alterations were observed with PANSS total or negative symptom scores or the individual hallucination/delusion items, or with neuropsychological deficit (BACS scores) or daily antipsychotic dose in probands (in chlorpromazine equivalents) (*P* > 0.05).

In nonpsychotic relatives, no significant correlations were observed between any altered network metric with BACS data or the presence of psychosis spectrum personality features. The nodal degree (*r* = −0.34, *P* < 0.001) and nodal efficiency *(r* = −0.38, *P* < 0.001) measures in the right MFG showed significant age-related decline, but these relations did not differ significantly from those of healthy controls (Supplementary Materials).

## Discussion

By investigating single-subject graphs reflecting the structure of gray matter network morphology in individuals with psychotic disorders and their first-degree relatives, we found that psychotic probands demonstrated clinically relevant decreased nodal efficiency in bilateral temporal lobes, mainly in the superior temporal cortex and temporal poles, relative to healthy individuals. Comparison of patients with their own nonpsychotic relatives revealed similar findings and an additional network alteration in the left thalamus. Nonpsychotic relatives, compared with community controls, showed lower nodal degree in the right prefrontal cortex, left pallidum, and left hippocampus, and higher nodal degree in the left posterior cingulate cortex and left thalamus with mostly parallel alterations of nodal efficiency in these regions. These findings in a study conducted by a multisite consortium with rigorous attention to consistency of image acquisition and clinical assessment provides evidence for the disruptions of superior temporal cortex in case probands but not in unaffected relatives, with minimal familiality of these alterations, suggesting that they may represent disease-related traits associated with psychotic illness. Altered anatomic connection mainly involving the prefrontal cortex, hippocampus, and striatum in relatives may be related to illness risk.

### Gray matter network alterations in probands

Network disorganization involving the superior and middle temporal cortex was observed in patients in comparison with their first-degree relatives and community controls. This alteration was associated with the level of residual psychosis symptom severity in our clinically stable psychosis proband cohort. Superior temporal regions include primary and secondary auditory cortices and areas related to language processing, while MTG is involved in semantic memory and language processes [54, 55]. These regions have been reported to have reduced local functional connectivity or abnormal network hubs in psychotic individuals using graph analysis [17, 56, 57]. Regional abnormalities in the superior temporal cortex have also been reported with both functional [20] and structural analysis [58, 59] in patients with psychotic disorders, which have been related to positive symptoms [59], including auditory hallucinations [60, 61]. These findings suggest an important role of the superior temporal cortex in the neuropathology of psychotic disorders.

It is important to emphasize that our findings of abnormal brain network organization in the superior temporal cortex reflect an alteration in its morphometric characteristics in relation to those of other brain areas. Our supplementary node-to-node correlational studies revealed that the most notable and consistent factors influencing altered nodal centrality of superior temporal regions involved alterations in its anatomic relationship with features of the prefrontal cortex and subcortical regions, including amygdala and striatum. Regional structural abnormalities and functional disconnectivity within frontotemporal circuits have been reported in psychotic illnesses in several studies [4, 62–64], and an altered relationship between temporal lobe function and striatal dopamine storage/synthesis capacity has been related to psychosis vulnerability [65]. Together with previous evidence from structural similarity and covariation network analyses in nonaffective psychoses [23, 27, 66], our findings support the notion of clinically relevant network hub abnormalities of superior temporal regions in psychotic disorders.

### Gray matter network alterations in relatives

In nonpsychotic first-degree relatives, we observed lower nodal efficiency in the right prefrontal cortex and left hippocampus and pallidum compared with healthy controls, suggesting a reduced use or capacity of these regions for propagating information to other nodes. These results parallel previous findings of lower clustering coefficient values and regional gray matter deficits in right frontal regions [66, 67], as well as functional and structural abnormalities in the hippocampus [68, 69], in individuals with familial risk for psychotic disorders. The impaired functional integration between the posterior hippocampus and prefrontal cortex has also been found in individuals at high risk for psychosis [70].

Nonpsychotic relatives also showed higher nodal centrality metrics in the left posterior cingulate cortex and left thalamus relative to healthy controls. Higher nodal centrality metrics suggest an enhanced connection of the node with others in the brain network configuration. Recent work investigating graph topology in relatives of SZ patients also indicated a higher nodal efficiency in the posterior cingulate cortex [71]. In a previous functional study defining gray matter “connections” using volumetric covariation, increased gray matter correlations with the posterior cingulate cortex were found in family members of SZ patients [29]. While previous gray matter volumetric analysis with B-SNIP participants indicated no significant abnormalities in nonpsychotic relatives [4], our network-based analysis may be more sensitive to patterns of gray matter changes, supporting the idea that anatomic graph analysis represents a promising approach for characterizing subtler neuroanatomic alterations associated with risk for psychotic disorders in the context of whole-brain anatomic configuration.

### Gray matter network differences between probands and relatives

The pattern of altered anatomic association of the superior temporal cortex in brain networks of probands contrasts with primarily cortical involvement of the frontal lobe in relatives. This represents a significant novel finding. In an investigation using a similar analytic approach, robust gray matter network-based changes in temporal regions were also not seen in relatives of schizophrenia probands [66]. Prior studies performing regional measurements of superior temporal cortex anatomy in unaffected siblings of patients with psychotic disorder also have not found evidence of superior temporal alterations [72, 73]. Thus, alterations in brain network organization involving superior temporal cortex appears to be associated with neuropathological manifestations of psychotic illness, while altered connection of the frontal cortex into brain networks in relatives may be related to familial illness risk.

This pattern of findings requires consideration of why a putative illness risk marker in unaffected relatives would be less robustly expressed in affected patients. While there is much evidence of frontal lobe alteration in schizophrenia, it is possible that illness-related changes in the temporal cortex and its connections with the frontal cortex as observed in our patients, or drug treatment effects on frontostriatal circuitry [74, 75], may variably affect frontal lobe connectivity in patients, reducing a more consistent pattern of frontal lobe connectivity alteration seen in nonpsychotic family members. While mechanisms for this difference between patients and unaffected relatives requires further study, our findings are consistent with the view that changes in STG are more related to being affected with a psychotic disorder than to familial factors, and thus may better serve as an illness than a risk biomarker.

### DSM diagnosis and B-SNIP biotype-specific analyses

In exploratory analysis, while patients with different DSM diagnoses did not differ in network metrics, changes in patients with SZ replicated all the network alterations as seen in the combined sample, while BD patients did not demonstrate any significant alteration. These analyses support continuum models for brain alterations across psychotic disorders, in which SZ and BD stand at the distant ends of a severity continuum of imaging deficits [1, 3], consistent with previous structural [4, 33] and resting-state fMRI [76] studies of regional brain features. A similar pattern of dimensional effects across disorders has been seen in heritability estimates of susceptibility genes [77], neuropsychological deficits [31], and clinical ratings [35, 78]. Network changes of patients with different B-SNIP biotypes were also in line with previous observations using other MR metrics, indicating Biotype 1 patients present with the most severe brain alterations while Biotype 3 patients have minimal brain alterations [3].

### Limitations

There are potential limitations that need to be considered when interpreting the current findings. First, this study employed anatomical templates to ensure the same number of nodes across individuals and enforce identical connectivity density to facilitate network comparisons, as in most previous studies. While this has limitations, we note that graphs for participants were analyzed in their native space, preserving inter-individual variability. Second, the potential confounding effects of antipsychotic medication on brain measures cannot be ruled out, though correlations of our findings with current antipsychotic drug dose were not significant. Third, although the spatial resolution of our data is comparable with that used in previous gray matter network analyses [36, 79, 80], higher resolution data acquisition in the future may increase precision of findings. Fourth, in the case of relatives, it is unclear whether the alterations reflect familial pathology or are linked to resilience and the absence of psychosis in this population. Prospective studies are needed to resolve this issue. Fifth, some previous studies adopted morphometric variables obtained from both T1-weighted imaging and diffusion-weighted imaging to map morphometric similarity networks [23, 25]. Future work with multimodal MRI may ultimately provide more precise morphometric network characterization as strategies for bringing these approaches into spatial alignment are better developed. Sixth, while our findings highlight the importance of STG and temporal pole alterations in anatomic network alterations in psychotic disorders, their relation to functional STG network integration remains to be determined. Finally, psychosis diagnoses were limited to common psychotic disorders, including SZ, SAD, and psychotic BD, not the full psychosis spectrum, and imaging studies were completed outside of acute episodes, potentially limiting clinical–pathological correlations.

## Funding and disclosure

This work was supported by the National Institute of Mental Health Grant Nos. MH077851 (to CAT), MH078113 (to MSK), MH077945 (to GDP), MH077852 (to Gunvant K. Thaker), and MH077862 (to JAS). The National Institute of Mental Health had no further role in study design; in the collection, analysis, and interpretation of the data; in the writing of the report; and in the decision to submit the paper for publication. The work was also supported by the National Natural Science Foundation of China (81671664, 81820108018, and 81621003), Miaozi Project in Science and Technology Innovation Program of Sichuan Province and 1.3.5 Project for Disciplines of Excellence, West China Hospital, Sichuan University (Project No. ZYYC08001, ZYJC18020). CAT has served on the advisory board for drug development for Intra-Cellular Therapies, Inc., as an ad hoc consultant for Eli Lilly, Sunovion, Astellas, Pfizer, and Merck, has been a council member and unpaid volunteer for the National Alliance on Mental Illness, and served as deputy editor for the American Psychiatric Association. MSK has received research support from Sunovion and GlaxoSmithKline. Remaining authors report no competing interests.

## Supplementary information

supplementary materials
