# Supplementary material for: Brain gray matter network organization in psychotic disorders
Source: Neuropsychopharmacology. 2019 Dec 7;45(4):666–74. doi: 10.1038/s41386-019-0586-2 (PMC7021697; doi:10.1038/s41386-019-0586-2)
Supplement: Supplementary file 1 — supplementary materials [file 41386_2019_586_MOESM1_ESM.docx]

**SUPPLEMENTAL MATERIALS**

Gray Matter Network Organization in Psychotic Disorders

**1. B-SNIP Brain Imaging Scanning Parameters**

Whole-brain structural T1-weighted images were acquired for all participants with a volumetric three-dimensional Magnetization Prepared Rapid Gradient Echo (MPRAGE) or Inversion Recovery-Prepared Spoiled Gradient-Echo (IR-SPGR) sequences, as appropriate for scanner brands, using 3.0T MRI scanners. The Alzheimer’s Disease Neuroimaging Initiative (ADNI) protocol (http://www.loni.ucla.edu/ADNI/Research/Cores/) and scans of traveling healthy individuals scanned across sites were used to enhance image consistency across all Bipolar and Schizophrenia Network for Intermediate Phenotypes (B-SNIP) sites. To ensure between- and within-site data compatibility, standardized ADNI phantoms at each site were imaged regularly, and drift in linear gradient calibration was monitored throughout the study. Measurements of non-linear geometric fidelity and correction for gradient non-linearity occurred once at each site, and again in the event of a hardware upgrade. The Magnetization Prepared Rapid Gradient Echo (MPRAGE) acquisition parameters were comparable across sites and details are presented in the Table S2.

**2. Imaging Quality Ratings**

Before data preprocessing, T1-weighted brain images of all subjects were inspected by two independent neuroradiologists without knowledge of participant characteristics, to ensure that there were no scanning artifacts or gross anatomic abnormalities in the participants that were included in statistical analyses. Furthermore, the image data quality was measured quantitively with the Computational Anatomy Toolbox (CAT, version 12.6, http://www.neuro.uni-jena.de/cat/), and the imaging quality ratings (IQR) of individual scans were obtained. An analysis of variance (ANOVA) was used to compared the IQR among patient probands (mean ± SD=84.10 ± 1.30), their first-degree relatives (mean ± SD=84.10 ± 1.45) and healthy control (mean ± SD =84.31 ± 1.11), and no significant differences in IQR were identified among them (F=1.95, p=0.14).

**3. Extraction of Brain Networks**

Single-subject gray matter networks were obtained for each participant based on similarity of cortical gray matter patterns using a completely automated and data-driven method that has been previously described [[1](#_ENREF_1)]. The method starts with defining the network’s nodes as regions of interest (ROI) corresponding to 3×3×3 mm^3^ voxel cubes, and their “connection” refers to edges indicating statistically similar gray matter morphology of two cubes, which is determined with correlation coefficients. As two similar cubes could be located at an angle from each other, the maximum correlation value over different rotations of the seed cube was used to estimate node similarity. Next, Weighted graphs are constructed after determining a threshold for each individual graph with a permutation-based method to ensure a significant similarity (p<0.05) for all individuals [[2](#_ENREF_2)]. Only positive similarity values survived this threshold.

Since similarity-based gray matter networks obtained using these procedures have different sizes, and typical network properties that vary with network size [[3](#_ENREF_3)], we normalized the gray matter networks using the methodology proposed by Batalle et al [[4](#_ENREF_4)] based on the unified Automated Anatomical Labeling (AAL) parcellation template [[5](#_ENREF_5)] and Statistical Parametric Mapping software (SPM, http://www.fil.ion.ucl.ac.uk/spm). After this procedure, all subjects had the same number of nodes, corresponding to the 90 brain regions of the AAL atlas. To perform the normalization, each cube in the similarity-based network was linked to the brain region in the AAL atlas to which most of the voxels in each cube belonged. Each pair of nodes was considered connected with a weight corresponding to the ratio of the sum actual significant correlations by the total possible connections between all the previous similarity-based network nodes belonging to the two ROIs. The weight obtained is bounded between 0 and 1. Self-connections were excluded. Finally, a 90×90 weighted normalized network of brain anatomic structures was obtained for each subject.

**4. Network Metrics**

Among the network metrics that were calculated, the C_p_ is a measure of the local interconnectivity of the network while L_p_ quantifies the ability for information propagation in the network, measuring segregation and integration of networks respectively. Here, L_p_ was calculated as the “harmonic mean” distance between all possible pairs of regions to address the disconnected graphs dilemma. A small-world network is characterized by high clustering and low path length [[6](#_ENREF_6)]. It has a similar characteristic path length, but higher clustering coefficient than a random network (which is based on the original output 90×90 random matrix and 100 randomized networks were used), i.e., normalized clustering coefficient (gamma, γ) = C_p_/C_random_ > 1, normalized characteristic path length (lambda, λ) = L_p_/L_random_ ≈ 1, and the small-worldness scalar (sigma, σ) = γ/λ larger than 1.

Network efficiency parameters including local efficiency (E_loc_) and global efficiency (E_glob_), which measure the capability of the network with regard to information transmission at the global and local levels [[7](#_ENREF_7)], were also calculated. Nodal centrality metrics including degree, efficiency and betweenness were also examined. Nodal degree represents the number of links connected to a node, whilst nodal betweenness represents the fraction of all shortest paths in the network that pass through a node [[6](#_ENREF_6)]. Nodal efficiency measures the ability of a node to propagate information with other nodes in a network [[8](#_ENREF_8)]. These parameters measure the importance of a node within a network from different aspects.

**5. Subnetwork Analysis**

Three main networks including default mode network (DMN), central executive network (CEN) and salience network (SN) that have been shown altered in psychosis probands and their relatives, typically in functional network analysis [[9](#_ENREF_9),[10](#_ENREF_10),[11](#_ENREF_11),[12](#_ENREF_12)], were constructed and their topological network metrics were calculated and compared among participant groups (see Table S3 for regions within each network). During the inter-group comparisons, analysis of covariance (ANCOVA) was performed to identify significant changes of the topological network metrics of selected networks in psychosis probands and their nonpsychotic relatives relative to healthy controls. Site, age, sex, race and handedness were included as covariates, and group differences in ANCOVA were corrected using false discovery rate (FDR) with a corrected p<0.05.

However, in the present study, no significant differences were identified in the topological metrics of CEN, DMN or SN in the psychosis probands or their nonpsychotic relatives in comparisons with healthy controls after correction for multiple comparisons (p>0.05, FDR corrected).

**6. Network Matrix Comparisons of Abnormal Nodes**

Alteration of regional nodal metrics in probands or relatives indicates an alteration of similarity with other nodes, as defined by structural correlation coefficients. In a secondary analysis, we thus compared the network correlation matrix (Fisher’s z-transformed) of aberrant nodes between psychotic probands, their nonpsychotic relatives and healthy controls to identify the specific gray matter correlation alterations associated with nodes with altered metrics with the network-based statistics (NBS) method [[13](#_ENREF_13)]. First, the nodes that exhibited significant inter-group differences in at least one of the three nodal centralities (node degree, efficiency and betweenness) were chosen. Then, a subset of connection matrices was created for each participant connecting with these altered nodes. Finally, the NBS approach was applied to define a set of suprathreshold links that connected with the abnormal nodes and survived from the FDR correction. A detailed description of this approach has been given previously [[13](#_ENREF_13)].

Four aberrant nodes were found in probands, including left superior temporal gyrus (STG), left middle temporal gyrus (MTG) and bilateral superior temporal pole (STP). In the matrix comparison of these nodes between probands and healthy controls, patient probands showed significant inter-group differences in gray matter associations between **left STG** and prefrontal cortex (left superior frontal gyrus and left rectus gyrus), subcortical regions (including left amygdala, right pallidum and right parahippocampal gyrus), bilateral insula, and right precuneus. Regions exhibiting differences in gray matter associations with **left MTG** mainly located in bilateral insula, left rectus gyrus and right amygdala. Regions exhibiting differences in gray matter associations with **left STP** mainly located in prefrontal regions (bilateral middle frontal gyrus and left rectus gyrus). For **right STP**, differences in the gray matter correlations were found mainly with frontal regions (including left superior frontal gyrus, left rectus gyrus, bilateral middle frontal gyrus, bilateral inferior frontal gyrus, and bilateral precentral gyrus), bilateral precuneus, right amygdala, right cuneus and left insula. All the findings survived the FDR correction. Details are presented in Table S4.

In nonpsychotic relatives, the regions exhibiting inter-group differences in gray matter associations (relatives vs. controls) with abnormal nodes were presented in Table S5.

**7. Within Family Correlations**

To explore potential familial patterns of brain network organization, analyses were conducted to examine the correlation of data from patients and their relatives from the same families across the full sample, and secondly within diagnoses for all metrics in which differences were seen in relatives or probands vs. the healthy controls. All analyses were performed after controlling site, age, sex, race and handedness. To similarly weight each family regardless of the number of family member participants, network parameters were averaged for unaffected family members related to the same proband. The FDR correction was used to correct for multiple hypothesis testing in the whole group analysis, and also in the secondary proband diagnosis specific analyses.

In altered metrics of patient probands, significant but moderate positive association between nodal parameters of patients and relatives was observed only in nodal efficiency of left STG (r=0.25, p=0.009). In abnormal network metrics of relatives, significant positive within-family associations were observed in nodal degree of left thalamus (r=0.39, p<0.001), and nodal efficiency of left hippocampus (r=0.23, p=0.018) and left thalamus (r=0.41, p<0.001).

The analyses were also conducted with regard to each DSM disorder separately. In patients with schizophrenia (SZ) and their relatives, positive associations were observed in nodal efficiency of left thalamus (r=0.44, p=0.017). Patients with schizoaffective disorder (SAD) and their relatives showed associations in nodal degree (r=0.55, p=0.002) and nodal efficiency (r=0.58, p=0.001) of left thalamus. Patients with psychotic bipolar disorder (BD) and their relatives did not show significant association in any of altered nodal network metrics after FDR correction.

**8.** **Age-related Analysis of Altered Network Metrics in Probands and Relatives**

Given the growing interests in aging effects on imaging phenotypes in psychotic disorders suggesting accelerated aging in patients with psychotic disorders [[14](#_ENREF_14),[15](#_ENREF_15)], we performed a linear regression analysis to characterize associations between altered nodal centrality metrics and age in probands and relatives respectively, and compared with those of healthy controls to identify inter-group differences in age-related changes of altered network metrics.

In psychosis probands, the altered nodal efficiency in left STG (r=-0.13, p=0.02), left MTG (r=-0.25, p<0.001) and bilateral STP (left: r=-0.24, p<0.001; right: r=-0.22, p<0.001) showed significant age-related decreases. In healthy controls, the nodal efficiency in left MTG (r=-0.36, p<0.001) and left STP (r=-0.18, p=0.10) significantly decreased with age. Comparisons of all the four regression models indicated no significant differences in age-related effects between probands and healthy controls.

Among regions that showed significantly altered nodal centrality metrics in nonpsychotic relatives, nodal degree (r=-0.34, p<0.001) and nodal efficiency (r=-0.38, p<0.001) of right middle frontal gyrus (MFG) had significant age-related decline with age. While for healthy controls, nodal degree (r=-0.39, p<0.001) and nodal efficiency (r=-0.45, p<0.001) of right MFG, and nodal efficiency of left hippocampus (r=-0.20, p=0.005) also had significant age-related decrease. Comparisons of these three regression models indicated no significant differences in age-related effects between relatives and healthy controls.

**9. Comparisons of Altered Network Metrics Across Patients with Different DSM Diagnoses**

In order to examine the extent of gray matter network changes in different diagnostic groups, comparisons between individuals diagnosed with SZ, SAD or psychotic BD and healthy controls were each conducted in network metrics showing significant changes in the whole proband group. Site, age, sex, race and handedness were included as covariates, and multiple comparison correction was conducted with the FDR procedure.

In contrast to healthy controls, individuals with SZ showed lower nodal efficiency in left STG, left MTG and bilateral STP as seen in the combined patient sample (FDR corrected p<0.05). Probands with SAD exhibited lower nodal efficiency in the left STG and left MTG relative to healthy controls (FDR corrected p<0.05). Probands with BD did not show any significant difference when compared to healthy subjects (p>0.05, FDR corrected). Details are presented in Table S6.

To examine inter-group differences of gray matter network topological metrics, we compared the network metrics among the probands with different DSM diagnoses at both global and nodal level. These analyses were conducted via ANCOVA and with the same covariates listed just above, but no significant diagnostic differences were observed.

While significant findings were not detected, consistent with our prior work, a pattern of significant deficits was observed with SZ and BD at the distant ends of a severity continuum and SAD being in the middle [[16](#_ENREF_16),[17](#_ENREF_17)]. This pattern of continuum effect is generally consistent with previous imaging studies from the B-SNIP consortium, including structural [[18](#_ENREF_18),[19](#_ENREF_19)] and resting-state fMRI findings [[20](#_ENREF_20)], and has been seen in heritability estimates of susceptibility genes [[21](#_ENREF_21)], neuropsychological deficits [[22](#_ENREF_22)] and clinical ratings [[23](#_ENREF_23),[24](#_ENREF_24),[25](#_ENREF_25)]. While deficits as a rule are greater in schizophrenia patients, there is a high level of overlap across diagnoses in all of these parameters, and thus we and others have begun examining patients with psychotic disorders as a group, seeking to identify subgroups of psychotic disorders empirically based on neurobiological rather than behavioral criteria [[26](#_ENREF_26)].

**10. Comparisons of Altered Network Metrics Across Patients with Different B-SNIP Biotypes**

Our previous studies have identified three distinct psychosis biotypes defined using neurophysiological and cognitive features, not including brain imaging data [[26](#_ENREF_26)]. In the present study, we compared gray matter network organization in individuals with each Biotype in exploratory analyses. Following similar comparison procedures as conducted above, inter-group comparisons between patient subtypes and healthy controls were conducted with altered metrics across the three Biotypes. Among all the probands included in our study, 65 subjects were of Biotype 1, 88 subjects were of Biotype 2 while 128 subjects were of Biotype 3. Forty-five remaining probands did not have complete biomarker data required to assign them a biotype.

In comparison with healthy controls, individuals of Biotype 1 exhibited lower nodal efficiency in the left STG, left MTG and bilateral STP (FDR corrected p<0.05). Probands of Biotype 2 exhibited lower nodal efficiency in the left STG relative to healthy controls (FDR corrected p<0.05). Biotype 3 cases exhibited lower nodal efficiency in the right STP in comparison with healthy individuals (FDR corrected p<0.05). In direct comparison of patients with different Biotypes, no significant findings were observed. Details are presented in Table S7.

**11. Comparisons of Altered Network Metrics Across Relatives of Individuals with Different DSM Diagnoses or BSNIP Biotypes**

Pair-wise comparisons of nodal metrics shown to be altered in the total relative group were conducted between high-risk individuals for each DSM diagnosis and healthy controls. Details are in Table S8.

Similar analyses were also conducted in each relative subgroup of patients with different Biotypes. Pair-wise comparisons of nodal metrics shown to be altered in the total relative group were conducted between unaffected relatives classified by their Biotype (Biotype 1, n=66; Biotype 2, n=79; Biotype 3, n=120) and healthy controls. Details are in Table S9.

**References**

1. Tijms BM, Series P, Willshaw DJ, Lawrie SM. Similarity-based extraction of individual networks from gray matter MRI scans. Cereb Cortex. 2012;22:1530-41.

2. Weese J, Rosch P, Netsch T, Blaffert T, Quist M. Gray-value based registration of CT and MR images by maximization of local correlation. In: Taylor C, Colchester A, editors. Medical Image Computing and Computer-Assisted Intervention, Miccai'99, Proceedings. 1999. p. 656-63.

3. van Wijk BCM, Stam CJ, Daffertshofer A. Comparing Brain Networks of Different Size and Connectivity Density Using Graph Theory. Plos One. 2010;5.

4. Batalle D, Munoz-Moreno E, Figueras F, Bargallo N, Eixarch E, Gratacos E. Normalization of similarity-based individual brain networks from gray matter MRI and its association with neurodevelopment in infants with intrauterine growth restriction. Neuroimage. 2013;83:901-11.

5. Tzourio-Mazoyer N, Landeau B, Papathanassiou D, Crivello F, Etard O, Delcroix N, et al. Automated anatomical labeling of activations in SPM using a macroscopic anatomical parcellation of the MNI MRI single-subject brain. Neuroimage. 2002;15:273-89.

6. Rubinov M, Sporns O. Complex network measures of brain connectivity: uses and interpretations. Neuroimage. 2010;52:1059-69.

7. Latora V, Marchiori M. Efficient behavior of small-world networks. Phys Rev Lett. 2001;87:17.

8. Achard S, Bullmore E. Efficiency and cost of economical brain functional networks. PLoS Comput Biol. 2007;3:e17.

9. Menon V. Large-scale brain networks and psychopathology: a unifying triple network model. Trends Cogn Sci. 2011;15:483-506.

10. Patel R, Spreng RN, Shin LM, Girard TA. Neurocircuitry models of posttraumatic stress disorder and beyond: a meta-analysis of functional neuroimaging studies. Neurosci Biobehav Rev. 2012;36:2130-42.

11. Buckner RL, Andrews-Hanna JR, Schacter DL. The brain's default network: anatomy, function, and relevance to disease. Ann N Y Acad Sci. 2008;1124:1-38.

12. Gusnard DA, Raichle ME, Raichle ME. Searching for a baseline: functional imaging and the resting human brain. Nat Rev Neurosci. 2001;2:685-94.

13. Zalesky A, Fornito A, Bullmore ET. Network-based statistic: identifying differences in brain networks. Neuroimage. 2010;53:1197-207.

14. Chiapponi C, Piras F, Fagioli S, Piras F, Caltagirone C, Spalletta G. Age-related brain trajectories in schizophrenia: a systematic review of structural MRI studies. Psychiatry Res. 2013;214:83-93.

15. Zhang W, Deng W, Yao L, Xiao Y, Li F, Liu J, et al. Brain Structural Abnormalities in a Group of Never-Medicated Patients With Long-Term Schizophrenia. Am J Psychiatry. 2015;172:995-1003.

16. Kempf L, Hussain N, Potash JB. Mood disorder with psychotic features, schizoaffective disorder, and schizophrenia with mood features: trouble at the borders. Int Rev Psychiatry. 2005;17:9-19.

17. Lake CR, Hurwitz N. Schizoaffective disorder merges schizophrenia and bipolar disorders as one disease--there is no schizoaffective disorder. Curr Opin Psychiatry. 2007;20:365-79.

18. Ivleva EI, Bidesi AS, Keshavan MS, Pearlson GD, Meda SA, Dodig D, et al. Gray matter volume as an intermediate phenotype for psychosis: Bipolar-Schizophrenia Network on Intermediate Phenotypes (B-SNIP). Am J Psychiatry. 2013;170:1285-96.

19. Ivleva EI, Clementz BA, Dutcher AM, Arnold SJM, Jeon-Slaughter H, Aslan S, et al. Brain Structure Biomarkers in the Psychosis Biotypes: Findings From the Bipolar-Schizophrenia Network for Intermediate Phenotypes. Biol Psychiatry. 2017;82:26-39.

20. Meda SA, Clementz BA, Sweeney JA, Keshavan MS, Tamminga CA, Ivleva EI, et al. Examining Functional Resting-State Connectivity in Psychosis and Its Subgroups in the Bipolar-Schizophrenia Network on Intermediate Phenotypes Cohort. Biol Psychiatry Cogn Neurosci Neuroimaging. 2016;1:488-97.

21. Cardno AG, Rijsdijk FV, Sham PC, Murray RM, McGuffin P. A twin study of genetic relationships between psychotic symptoms. Am J Psychiatry. 2002;159:539-45.

22. Hill SK, Reilly JL, Keefe RS, Gold JM, Bishop JR, Gershon ES, et al. Neuropsychological impairments in schizophrenia and psychotic bipolar disorder: findings from the Bipolar-Schizophrenia Network on Intermediate Phenotypes (B-SNIP) study. Am J Psychiatry. 2013;170:1275-84.

23. Tamminga CA, Ivleva EI, Keshavan MS, Pearlson GD, Clementz BA, Witte B, et al. Clinical phenotypes of psychosis in the Bipolar-Schizophrenia Network on Intermediate Phenotypes (B-SNIP). Am J Psychiatry. 2013;170:1263-74.

24. Mancuso SG, Morgan VA, Mitchell PB, Berk M, Young A, Castle DJ. A comparison of schizophrenia, schizoaffective disorder, and bipolar disorder: Results from the Second Australian national psychosis survey. J Affect Disord. 2015;172:30-7.

25. Keshavan MS, Morris DW, Sweeney JA, Pearlson G, Thaker G, Seidman LJ, et al. A dimensional approach to the psychosis spectrum between bipolar disorder and schizophrenia: the Schizo-Bipolar Scale. Schizophr Res. 2011;133:250-4.

26. Clementz BA, Sweeney JA, Hamm JP, Ivleva EI, Ethridge LE, Pearlson GD, et al. Identification of Distinct Psychosis Biotypes Using Brain-Based Biomarkers. Am J Psychiatry. 2016;173:373-84.

**Table 1**. Demographic and clinical parameters for patients of each diagnosis, their nonpsychotic relatives and healthy controls.

|  | **SZ**  **(N=107)** | **SAD**  **(N=87)** | **BD**  **(N=132)** | **SZ-Rel**  **(N=120)** | **SAD-Rel**  **(N=83)** | **BD-Rel**  **(N=112)** | **HC**  **(N=202)** | F | *p* |
| --- | --- | --- | --- | --- | --- | --- | --- | --- | --- |
|  | **Mean (standard deviation)** | | | | | | |  |  |
| Age (years) | 34.41 (12.53) | 36.01 (11.69) | 35.67 (12.91) | 40.99 (15.22) | 39.57 (16.08) | 38.74 (15.98) | 36.59 (12.55) | 3.30 | 0.003 |
| Education | 12.96 (2.49) | 13.19 (2.25) | 14.36 (2.26) | 13.98 (2.37) | 14.42 (2.82) | 14.61 (2.76) | 14.96 (2.39) | 10.95 | <0.001 |
| GAF | 48.56 (12.12) | 47.56 (12.65) | 61.40 (12.38) | 76.70 (12.55) | 77.09 (12.79) | 77.46 (12.03) | 86.65 (6.54) | 226.59 | <0.001 |
| WRAT | 96.65 (15.85) | 99.18 (15.20) | 103.78 (13.76) | 96.87 (14.27) | 103.94 (17.05) | 103.91 (13.13) | 103.64 (14.10) | 6.47 | <0.001 |
| BACS | -1.67 (1.38) | -1.32 (1.38) | -0.72 (1.27) | -0.59 (1.15) | -0.32 (1.35) | -0.02 (1.10) | 0.02 (1.11) | 32.02 | <0.001 |
| PANSS_Total | 71.54 (17.23) | 70.76 (15.19) | 54.15 (13.70) | / | / | / | / | 47.97 | <0.001 |
| PANSS_Positive | 18.53 (5.30) | 18.94 (4.56) | 12.86 (4.34) | / | / | / | / | 59.64 | <0.001 |
| PANSS_Negative | 17.60 (6.02) | 15.69 (4.67) | 12.03 (3.95) | / | / | / | / | 39.38 | <0.001 |
| YMRS_Total | 7.01 (6.06) | 7.73 (5.96) | 5.18 (6.44) | / | / | / | / | 4.92 | 0.008 |
| MADRS_Total | 10.57 (8.66) | 14.64 (10.34) | 9.86 (8.66) | / | / | / | / | 7.66 | 0.001 |
| CPZ dose (mg/day) | 418.02 (307.73) | 517.14 (507.28) | 302.98 (302.11) | / | / | / | / | 5.39 | 0.005 |
|  | N (Percentage) | | | | | | | χ2 | p |
| Sex (male %) | 67 (62.6%) | 34 (39.1%) | 42 (31.8%) | 32 (26.7%) | 27 (32.5%) | 32 (28.6%) | 88 (43.6%) | 44.08 | <0.001 |
| Handed (Right %) | 89 (83.2%) | 76 (87.4%) | 108 (81.8%) | 103 (85.8%) | 74 (89.2%) | 95 (84.8%) | 177 (87.6%) | 3.85 | 0.697 |
| Race |  |  |  |  |  |  |  |  |  |
| Caucasian | 49 (45.8%) | 40 (46.0%) | 101 (76.5%) | 67 (55.8%) | 57 (68.7%) | 90 (80.4%) | 134 (66.3%) | 62.66 | <0.001 |
| African American | 49 (45.8%) | 42 (48.3%) | 24 (18.2%) | 45 (37.5%) | 24 (28.9%) | 17 (15.2%) | 50 (24.8%) |  |  |
| Other | 9 (8.4%) | 5 (5.7%) | 7 (5.3%) | 8 (6.7%) | 2 (2.4%) | 5 (4.5%) | 18 (8.9%) |  |  |

Abbreviations: SZ-schizophrenia, SAD-schizoaffective disorder, BD-psychotic bipolar disorder, BACS-Brief Assessment of Cognition in Schizophrenia (z-score), CPZ-Chlorpromazine Equivalent Antipsychotic Dosage, GAF-Global Assessment of Functioning, MADRS-Montgomery-Åsberg Depression Rating Scale, PANSS-Positive and Negative Syndrome Scale, WRAT-Wide-Range Achievement Test, YMRS-Young Mania Rating Scale.

**Table S2**. Scanner parameters of T1-weighted images across all B-SNIP sites.

| Sites | TR (ms) | TE (ms) | Flip angle (degree) | Slices (N) | Matrix (mm) | Voxel Size (mm) | Vendor |
| --- | --- | --- | --- | --- | --- | --- | --- |
| Baltimore | 6.80 | 2.91 | 9 | 160 | 256×240 | 1×1×1.2 | Siemens Trio |
| Boston | 7.0 | 3.00 | 8 | 166 | 256×256 | 1×1×1.2 | GE Signa HDxt |
| Chicago | 6.99 | 2.85 | 8 | 166 | 256×256 | 1×1×1.2 | GE Signa HDx |
| Detroit | 6.80 | 2.74 | 8 | 160 | 256×240 | 1×1×1.2 | Siemens Trio |
| Dallas | 6.80 | 3.10 | 8 | 170 | 256×240 | 1×1×1.2 | Philips Achieva |
| Hartford | 7.20 | 2.91 | 9 | 160 | 256×240 | 1×1×1.2 | Siemens Allegra |

**Table S3**. Regions within each selected network of the central executive network (CEN), default mode network (DMN) and salience network (SN) according to Automated Anatomical Labeling (AAL) parcellation template. All regions given below are bilateral.

| Central Executive Network  (CEN) | Default Mode Network  (DMN) | Salience Network  (SN) |
| --- | --- | --- |
| Dorsolateral superior frontal gyrus | Superior frontal gyrus, orbital part | Pars opercularis |
| Middle frontal gyrus | Medial superior frontal gyrus | Insula |
| Middle frontal gyrus, orbital part | Medial orbitofrontal gyrus | Anterior cingulate gyrus |
| Pars triangularis | Rectus gyrus | Amygdala |
| Pars orbitalis | Posterior cingulate gyrus | Putamen |
| Superior parietal gyrus | Hippocampus | / |
| Supramarginal gyrus | Parahippocampal gyrus | / |
| Caudate nucleus | Angular gyrus | / |
| / | Superior temporal gyrus | / |
| / | Superior temporal pole | / |
| / | Middle temporal gyrus | / |

**Table S4**. Regions showing significant differences in gray matter correlations with each abnormal node identified in probands in comparison with healthy controls.

| **Abnormal nodes in probands** | Regions showed abnormal gray matter correlations with each abnormal node (t value*) |
| --- | --- |
| L superior temporal gyrus | L insula (4.03)  L rectus gyrus (3.98)  L amygdala (3.14)  R pallidum (2.90)  L superior frontal gyrus, orbital part (2.79)  R parahippocampal gyrus (2.72)  R precuneus (2.69)  R insula (2.68) |
| L superior temporal pole | L rectus gyrus (2.96)  R middle frontal gyrus, orbital part (2.72)  L middle frontal gyrus, orbital part (2.66) |
| R superior temporal pole | L superior frontal gyrus, orbital part (4.18)  L rectus gyrus (3.96)  R precuneus (3.78)  L precentral gyrus (3.74)  R pars opercularis (3.68)  R cuneus (3.65)  L middle frontal gyrus (3.63)  R pars triangularis (3.58)  R middle frontal gyrus (3.51)  R precentral gyrus (3.44)  L middle frontal gyrus, orbital part (3.42)  R amygdala (3.40)  R middle frontal gyrus, orbital part (3.35)  L insula (3.26)  L pars triangularis (2.95)  L superior frontal gyrus (2.86)  L precuneus (2.73) |
| L middle temporal gyrus | L insula (5.03)  L rectus gyrus (3.55)  R insula (2.66)  R amygdala (2.51) |

^*^  t values that survived from false discovery rate (FDR) correction.

Abbreviation: L-left, R-right.

**Table S5**. Regions showing significant differences in gray matter correlation with each abnormal node identified in the nonpsychotic relatives in comparison with healthy controls.

| **Abnormal nodes in nonpsychotic relatives** | Regions showed abnormal gray matter correlations with each abnormal node (t value ^*^) |
| --- | --- |
| R middle frontal gyrus | L rectus gyrus (5.05)  L amygdala (3.94)  L caudate (3.07)  R precuneus (2.86) |
| R orbital inferior frontal gyrus | L rectus gyrus (5.77)  L amygdala (3.75)  R Cuneus (2.94) |
| L posterior cingulate gyrus | R postcentral gyrus (4.10)  R angular gyrus (4.03)  L putamen (3.46) |
| L hippocampus | L rectus gyrus (5.76)  L middle cingulate gyrus (3.32)  R middle cingulate gyrus (3.34) |
| L pallidum | R cuneus (4.56)  L amygdala (3.06)  R fusiform gyrus (2.83) |
| L thalamus | L rectus gyrus (5.66)  L amygdala (3.98) |

^*^  t values that survived from false discovery rate (FDR) correction.

Abbreviation: L-left, R-right.

**Table S6**. The pair-wise comparisons of nodal metrics shown to be altered in the total proband group between individuals with each DSM diagnosis and healthy controls.

| Areas with altered metrics | Altered nodal metric | Directionality | F | p ^#^ | Effect size  (partial η^2^) |
| --- | --- | --- | --- | --- | --- |
| ***Schizophrenia vs Healthy controls*** | |  |  |  |  |
| Superior temporal gyrus. L | Efficiency | Deceased | 15.11 | 0.001 | 0.048 |
| Superior temporal pole. L | Efficiency | Deceased | 12.10 | 0.002 | 0.039 |
| Superior temporal pole. R | Efficiency | Deceased | 14.21 | 0.001 | 0.045 |
| Middle temporal gyrus. L | Efficiency | Deceased | 13.05 | 0.001 | 0.041 |
| ***Schizoaffective disorder vs Healthy controls*** | |  |  |  |  |
| Superior temporal gyrus. L | Efficiency | Deceased | 7.73 | 0.012 | 0.027 |
| Middle temporal gyrus. L | Efficiency | Deceased | 8.69 | 0.008 | 0.030 |
| ***Bipolar disorder vs Healthy controls*** | |  |  |  |  |
| No significant findings | | | | | |

^#^  Corrected p values using false discovery rate (FDR).

Abbreviation: L-left, R-right.

**Table S7**. The pair-wise comparisons of nodal metrics shown to be altered in the total proband group between individuals with each Biotype and healthy controls.

| Areas with altered metrics | Altered nodal metric | Directionality | F | p ^#^ | Effect size  (partial η^2^) |
| --- | --- | --- | --- | --- | --- |
| ***Biotype 1 patients vs Healthy controls*** | |  |  |  |  |
| Superior temporal gyrus. L | Efficiency | Deceased | 9.90 | 0.015 | 0.037 |
| Superior temporal pole. L | Efficiency | Deceased | 6.29 | 0.026 | 0.024 |
| Superior temporal pole. R | Efficiency | Deceased | 8.72 | 0.015 | 0.032 |
| Middle temporal gyrus. L | Efficiency | Deceased | 8.61 | 0.015 | 0.032 |
| ***Biotype 2 patients vs Healthy controls*** | |  |  |  |  |
| Superior temporal gyrus. L | Efficiency | Deceased | 7.59 | 0.019 | 0.026 |
| ***Biotype 3 patients vs Healthy controls*** | |  |  |  |  |
| Superior temporal pole. R | Efficiency | Deceased | 6.81 | 0.023 | 0.021 |

^#^ Corrected p values using false discovery rate (FDR).

Abbreviation: L-left, R-right.

**Table S8**. The pair-wise comparisons of nodal metrics shown to be altered in the total relative group between relatives of individuals with each DSM diagnosis and healthy controls.

| Areas with altered metrics | Altered nodal metric | Directionality | F | p ^#^ | Effect size  (partial η^2^) |
| --- | --- | --- | --- | --- | --- |
| ***Relatives of schizophrenia vs Healthy controls*** | |  |  |  |  |
| R middle frontal gyrus | Degree | Decreased | 5.08 | 0.036 | 0.016 |
| R orbital inferior frontal gyrus | Degree | Decreased | 10.08 | 0.006 | 0.031 |
| L posterior cingulate gyrus | Degree | Increased | 8.75 | 0.008 | 0.027 |
| L hippocampus | Degree | Decreased | 9.90 | 0.006 | 0.031 |
| L pallidum | Degree | Decreased | 22.85 | <0.001 | 0.068 |
| L thalamus | Degree | Increased | 23.06 | <0.001 | 0.068 |
| R middle frontal gyrus | Efficiency | Decreased | 6.49 | 0.018 | 0.20 |
| R orbital inferior frontal gyrus | Efficiency | Decreased | 17.40 | <0.001 | 0.053 |
| L posterior cingulate gyrus | Efficiency | Increased | 8.50 | 0.009 | 0.026 |
| L hippocampus | Efficiency | Decreased | 7.30 | 0.013 | 0.023 |
| L thalamus | Efficiency | Increased | 21.02 | <0.001 | 0.063 |
| ***Relatives of schizoaffective disorder vs Healthy controls*** | | |  |  |  |
| R middle frontal gyrus | Degree | Decreased | 10.86 | 0.006 | 0.038 |
| R orbital inferior frontal gyrus | Degree | Decreased | 8.72 | 0.008 | 0.030 |
| L posterior cingulate gyrus | Degree | Increased | 10.81 | 0.006 | 0.037 |
| R middle frontal gyrus | Efficiency | Decreased | 7.60 | 0.012 | 0.27 |
| R orbital inferior frontal gyrus | Efficiency | Decreased | 7.18 | 0.013 | 0.025 |
| L posterior cingulate gyrus | Efficiency | Increased | 9.26 | 0.007 | 0.032 |
| ***Relatives of bipolar disorder vs Healthy controls*** | |  |  |  |  |
| R orbital inferior frontal gyrus | Degree | Decreased | 5.60 | 0.028 | 0.018 |
| L posterior cingulate gyrus | Degree | Increased | 10.13 | 0.006 | 0.032 |
| L hippocampus | Degree | Decreased | 10.39 | 0.006 | 0.033 |
| L pallidum | Degree | Decreased | 6.62 | 0.017 | 0.021 |
| R orbital inferior frontal gyrus | Efficiency | Decreased | 4.60 | 0.045 | 0.015 |
| L posterior cingulate gyrus | Efficiency | Increased | 7.66 | 0.012 | 0.024 |
| L hippocampus | Efficiency | Decreased | 7.40 | 0.013 | 0.024 |

^#^ Corrected p values using false discovery rate (FDR).

Abbreviation: L-left, R-right.

**Table S9**. The pair-wise comparisons of nodal metrics shown to be altered in the total relative group between relatives of each Biotype and healthy controls.

| Areas with altered metrics | Altered nodal metric | Directionality | F | p ^#^ | Effect size  (partial η^2^) |
| --- | --- | --- | --- | --- | --- |
| ***Relatives of Biotype 1 proband vs Healthy controls*** | | |  |  |  |
| L posterior cingulate gyrus | Degree | Increased | 5.89 | 0.034 | 0.022 |
| L pallidum | Degree | Decreased | 10.45 | 0.005 | 0.038 |
| L thalamus | Degree | Increased | 6.20 | 0.030 | 0.023 |
| R orbital inferior frontal gyrus | Efficiency | Decreased | 7.50 | 0.018 | 0.028 |
| L thalamus | Efficiency | Increased | 5.13 | 0.049 | 0.019 |
| ***Relatives of Biotype 2 probands vs Healthy controls*** | | |  |  |  |
| No significant findings | | | | | |
| ***Relatives of Biotype 3 probands vs Healthy controls*** | | |  |  |  |
| R middle frontal gyrus | Degree | Decreased | 8.23 | 0.013 | 0.025 |
| R orbital inferior frontal gyrus | Degree | Decreased | 17.56 | <0.001 | 0.053 |
| L posterior cingulate gyrus | Degree | Increased | 21.13 | <0.001 | 0.063 |
| L hippocampus | Degree | Decreased | 17.72 | <0.001 | 0.053 |
| L pallidum | Degree | Decreased | 17.04 | <0.001 | 0.051 |
| L thalamus | Degree | Increased | 12.57 | 0.018 | 0.038 |
| R middle frontal gyrus | Efficiency | Decreased | 6.23 | 0.030 | 0.19 |
| R orbital inferior frontal gyrus | Efficiency | Decreased | 17.84 | <0.001 | 0.054 |
| L posterior cingulate gyrus | Efficiency | Increased | 18.47 | <0.001 | 0.055 |
| L hippocampus | Efficiency | Decreased | 12.13 | 0.002 | 0.037 |
| L thalamus | Efficiency | Increased | 13.63 | 0.001 | 0.041 |

^#^ Corrected p values using false discovery rate (FDR).

Abbreviation: L-left, R-right.

Figure S1. Scatter plots of correlations between abnormal network metrics and clinical ratings in psychotic probands.


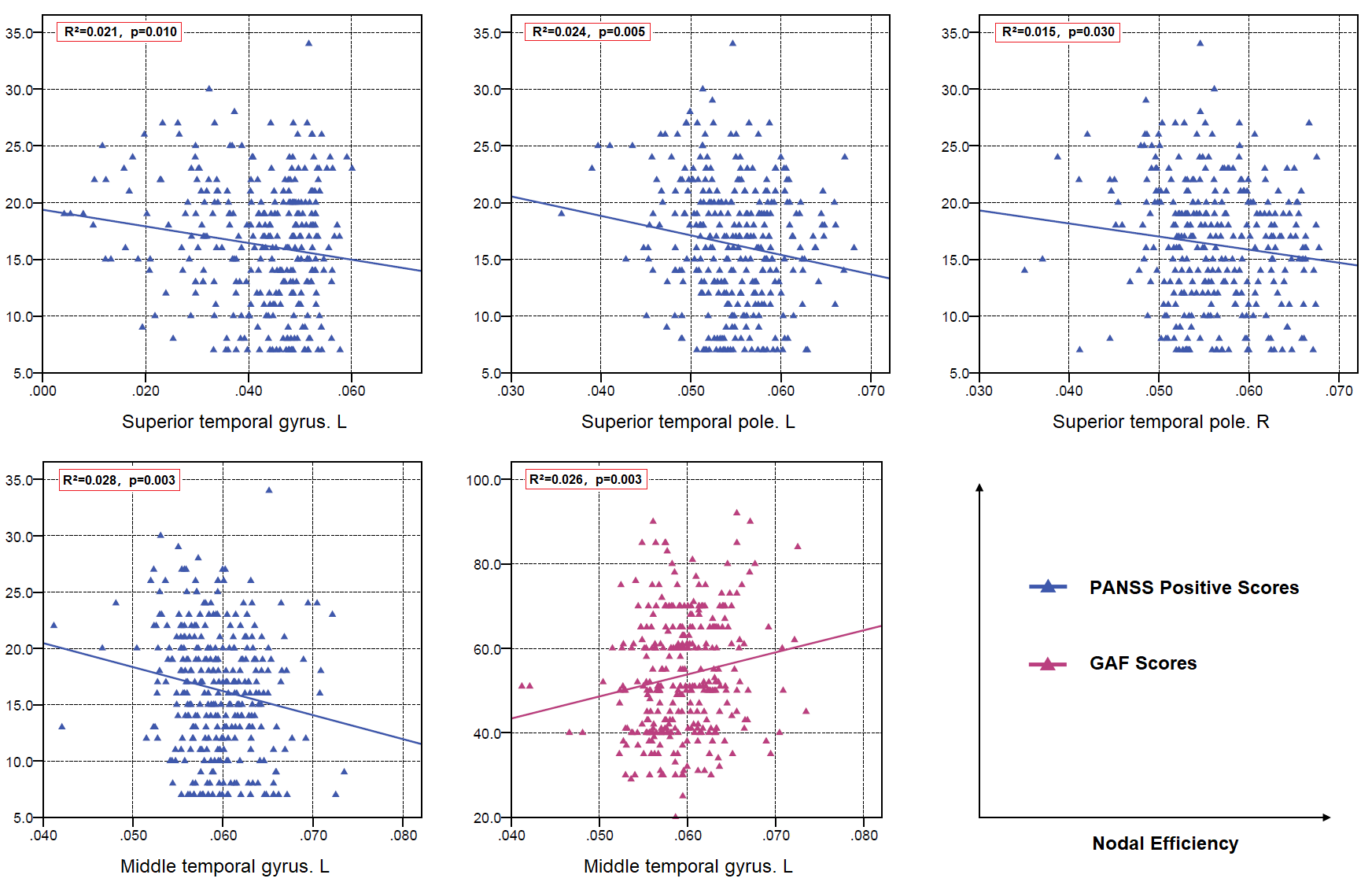


Abbreviations: L-left, R-right.
